# Refined Multiscale Entropy Using Fuzzy Metrics: Validation and Application to Nociception Assessment †

**DOI:** 10.3390/e21070706

**Published:** 2019-07-18

**Authors:** José F. Valencia, Jose D. Bolaños, Montserrat Vallverdú, Erik W. Jensen, Alberto Porta, Pedro L. Gambús

**Affiliations:** 1 Department of Electronic Engineering, Universidad de San Buenaventura, Cali 760033, Colombia; 2 Department of Automatic Control, Universitat Politècnica de Catalunya, 08028 Barcelona, Spain; 3 Center for Biomedical Engineering Research, Universitat Politècnica de Catalunya, 08028 Barcelona, Spain; 4 CIBER of Bioengineering, Biomaterials and Nanomedicine (CIBER-BBN), 08028 Barcelona, Spain; 5 Research and Development Department, Quantium Medical SL, 08302 Mataró, Spain; 6 Department of Biomedical Sciences for Health, University of Milan, 20133 Milan, Italy; 7 Department of Cardiothoracic-Vascular Anesthesia and Intensive Care, IRCCS Policlinico San Donato, San Donato Milanese, 20097 Milan, Italy; 8 Systems Pharmacology Effect Control & Modeling (SPEC-M) Research Group, Department of Anesthesia, Hospital CLINIC de Barcelona, 08036 Barcelona, Spain; 9 Department of Anesthesia and Perioperative Care, University of California San Francisco (UCSF), San Francisco, CA 94143, USA

**Keywords:** fuzzy entropy, conditional entropy, complexity, electroencephalography, pain assessment, refined multiscale entropy, sample entropy, sedation-analgesia

## Abstract

The refined multiscale entropy (RMSE) approach is commonly applied to assess complexity as a function of the time scale. RMSE is normally based on the computation of sample entropy (SampEn) estimating complexity as conditional entropy. However, SampEn is dependent on the length and standard deviation of the data. Recently, fuzzy entropy (FuzEn) has been proposed, including several refinements, as an alternative to counteract these limitations. In this work, FuzEn, translated FuzEn (TFuzEn), translated-reflected FuzEn (TRFuzEn), inherent FuzEn (IFuzEn), and inherent translated FuzEn (ITFuzEn) were exploited as entropy-based measures in the computation of RMSE and their performance was compared to that of SampEn. FuzEn metrics were applied to synthetic time series of different lengths to evaluate the consistency of the different approaches. In addition, electroencephalograms of patients under sedation-analgesia procedure were analyzed based on the patient’s response after the application of painful stimulation, such as nail bed compression or endoscopy tube insertion. Significant differences in FuzEn metrics were observed over simulations and real data as a function of the data length and the pain responses. Findings indicated that FuzEn, when exploited in RMSE applications, showed similar behavior to SampEn in long series, but its consistency was better than that of SampEn in short series both over simulations and real data. Conversely, its variants should be utilized with more caution, especially whether processes exhibit an important deterministic component and/or in nociception prediction at long scales.

## 1. Introduction

Recently, fuzzy entropy (FuzEn) has been proposed as an entropy measure that is more consistent and less dependent on the data length [1,2]. Indeed, FuzEn applys the concept of “fuzzy sets” and membership functions, introduced by Zadeh in 1965, to characterize input–output relations with stochastic components [3]. Some applications of FuzEn in biomedical signal processing has been presented in [4,5,6,7,8]. In order to improve the performance of FuzEn in short time series, several variations have been included in its computation. Some of them are centered and averaged fuzzy entropy [9] and inherent fuzzy entropy [10].

FuzEn, as sample entropy (SampEn) and other entropy rates, gives only a single scale representation of the behavior of a time series. However, these measurements can be extended to provide a multiscale assessment of irregularity of the time series. Refined multiscale entropy (RMSE), proposed in [11], is a technique that uses SampEn as an entropy-based measure in order to quantify the complexity of a time series in different time scales, which has been applied in processing of electrocardiogram (ECG) and electroencephalogram (EEG) signals [12,13,14,15]. Computation of RMSE is similar to multiscale entropy (MSE) [16] except for two significant modifications: (i) RMSE improves the procedure applied to remove the fast time scales in the signal, avoiding the aliasing, and; (ii) it modifies the coarse-graining procedure to avoid an artificial decrease of the entropy as the fast time scales in the signal are eliminated, which is caused by the reduction of the standard deviation that is generated by the filtering process.

Characterization of time series by means of RMSE requires relatively long length series, which increases the computation time and makes difficult the implementation of this algorithm in real time monitoring systems. The implementation of FuzEn and its variants, which offer an entropy-based measure that is less dependent on the data length, emerges as alternative to the traditional SampEn for the real-time multiscale analysis. This can be very useful, for example, to monitor patients, using physiological signals as the EEG in critical settings such as critical care units. For example, under sedation/anesthesia during surgery the assessment of complexity of the EEG via RMSE was found useful for monitoring the level of consciousness and preventing pain [15]. The assessment of EEG complexity as a function of time scales using of RMSE is motivated by the observation that the EEG contains oscillations at particular frequency bands, and these oscillations become slower and more regular at higher doses of intravenous anesthetic such as the propofol. In this sense, slower and less unpredictable oscillations can be associated with a deeper state of sedation [15].

The aim of this study is to compare the performance of SampEn and FuzEn metrics, with its different variants, when they are exploited for a multiscale analysis by means of RMSE. Synthetic and experimental time series are analyzed in order to evaluate the behavior of RMSE metrics in signals with different characteristics: stochastic without or with long-range correlation, stochastic but partially predictable, fully predictable determinist or chaotic. The study also involves the analysis of time series with different lengths and the application of RMSE to assess the prediction probability of pain response in patients under sedation-analgesia.

## 2. Methods 

### 2.1. Database 

#### 2.1.1. Synthetic Time Series

Synthetic signals were categorized in:

(a) Type-1, which included (i) Gaussian white noise (GWN) to simulate a fully unpredictable process; (ii) 1/f noise or pink noise (1/f) to generate a stochastic signal with long-range correlation; (iii) second-order autoregressive process (AR025), driven by GWN, to simulate a partially predictable stochastic process. The AR025 was shaped to have a power spectrum peak with central frequency at 0.25 cycles per sample and pole modulus *p* = 0.98. The parameters in AR025 were proposed to check the ability of RMSE to avoid aliasing when the downsampling procedure is applied [11]. Sixty realizations of 100,000 samples were generated for each process (GWN, 1/f, and AR025).
(b) Type-2, which included signals generated from (i) logistic map (LM), defined by $x_{n+1}=ax_{n}\left(1-x_{n}\right)$, where the parameter "a" controls the value of the samples such that the signals converges to a single fixed point if a≤3, oscillates if 3<a<3.54, and becomes increasingly chaotic and more and more complex structures emerge for larger a [17]. In this study, LM with values of the parameter a equals to 3.5 (oscillation condition, LM-3.5), 3.7 (chaotic condition, LM-3.7), and 3.9 (chaotic condition, LM-3.9) were considered; (ii) Henon map (HM), defined by $x_{n+1}=1-\alpha x_{n}{}^{2}+\beta x_{n-1}$ [18], was exploited to conduct more detailed exploration of the chaotic dynamics, using α=1.4 and β=0.3. Thirty realizations of 50,000 samples were generated for each process (LM-3.5, LM-3.7, LM-3.9, and HM).

#### 2.1.2. Experimental Time Series

The different approaches of RMSE were applied to EEG signals recorded from 378 patients under sedation-analgesia during an ultrasonographic endoscopy (USE) of the upper gastrointestinal tract. USE is a procedure with an approximate duration of 1 h, which includes periods of stability in the concentration of the anesthetic drugs, allowing the outcome of painful stimulus to be studied in relation with the level of sedation. This exploration required at least two endoscopy tube insertions: the first component carrying a regular gastroscope and a second component carrying the needle for biopsy. This study was approved by the Ethic Committee of Clinical Research of Hospital Clinic de Barcelona and all patients signed a written informed consent.

Patients were routinely monitored in the USE room. A single channel of the raw EEG signal was acquired using three electrodes: the positive electrode in the middle forehead; the negative electrode in the malar bone, and; the reference electrode in the left forehead. The recorded EEGs had an average duration of 60 m and were sampled at 900 samples per second with a resolution of 16 bits per sample. Propofol and remifentanil were infused as, respectively, anesthetic and analgesic agents by means of a target-controlled infusion system (FreseniusVial, Chemin de Fer, Béziers, France). The Ramsay sedation scale (RSS) score [19] was evaluated, by the attending anesthesiologist, at random times during the procedure. Random times were decided instead of a predefined schedule to avoid that factors associated with time, such as the infusion volume of propofol and remifentanil, could affect the results of the RSS measurements. Table 1 contains information about the annotated RSS scores in the database, which are between 2 and 6.

After the application of painful stimulation, two observed categorical responses were selected in the database (see Table 1): (i) the presence (RSS score between 2 and 5) or the absence (RSS = 6) of movement after nail bed compression, and; (ii) the presence or absence of gag reflex (GAG) after endoscopy tube insertion, where GAG = 1 corresponds to a positive nausea reflex, while GAG = 0 corresponds to no response after tube insertion. As the evaluation of RSS scores was done at random times, and the duration of every exploration was determined by the procedure per se, the number of RSS measurements were not equal in all patients (the median number of RSS score evaluations was 11 per patient). The number of GAG evaluations was between one and two per patient, according to the number of endoscopy tube insertion.

The next preprocessing steps were applied to EEG signals:

(i) They were resampled at 128 Hz after applying a band-pass finite impulse response (FIR) filter of 10th order with cut-off frequencies of 0.1–45 Hz, in order to limit the EEG signal to the traditional frequency bands: δ (0.1–4 Hz), θ (4–8 Hz), α (8–14 Hz), and β (14–30 Hz).
(ii) The filtered and resampled EEG signals were divided into windows of 1-min duration taken just before the response annotation according to RSS or GAG classification.
(iii) The 1-min EEG segments were associated to the correspondent annotated response (RSS or GAG) by considering that the sedation level should remain constant if the plasma concentration of remifentanil (CeRemi) and propofol (CeProp) remains unvaried. In this work, CeRemi and CeProp were considered constant if the variation of them (ΔCeRemi, ΔCeProp), between the first and the last second of the 1-min length window, was ΔCeRemi < 0.1 ng/mL and ΔCeProp < 0.1 µg/mL.
(iv) If CeRemi and CeProp were not constant during the 1-min length window, the window was maintained but cut at the sample where the conditions were satisfied. If the total useful length was less than 50 s the overall segment was excluded from the analysis.
(v) Windows of EEG were filtered with a filter based on the analytic signal envelope in order to reduce high-amplitude peaks of noise [20].
(vi) If the difference between adjacent samples was higher than 10% of the mean of the differences of the previous ten samples, the window was cut at the sample where the artifact was detected. If the total useful length was less than 50 s the overall segment was excluded from the analysis. After that, only the EEG windows with a duration between 50 and 60 s were included in the analysis.

Table 1 contains information about the number of EEG windows exploited in the present study for each annotated response.

### 2.2. SampEn and Fuzzy Approaches as Entropy Rates 

Let x={x(i),i=1,…,N} be a time series where i represents the number of the sample and N is the length of the series, any estimate of an entropy rate (rate of information generation) is based on a method for measuring the probability that two patterns of length *m*, $x_{m}\left(i\right)=(x\left(i\right),x\left(i-1\right),\ldots ,x(i-m+1))$ and $x_{m}\left(j\right)=\left(x\left(j\right),x\left(j-1\right),\ldots ,x\left(j-m+1\right)\right)$, that are similar in the *m*-dimensional phase-space continue being similar after adding a new sample in the pattern, i.e., $x_{m+1}\left(i+1\right)$ and $x_{m+1}\left(j+1\right)$ are also similar in the (m+1)-dimensional phase-space. In this sense, entropy rates allow the regularity of the time series x(i) to be quantified, showing high values for irregular or unpredictable series (series with low probability that two similar patterns $x_{m}\left(i\right)$ and $x_{m}\left(j\right)$ remain similar after adding new samples) and low values for regular or predictable series.

#### 2.2.1. SampEn

In SampEn [21], two patterns ($x_{m}\left(i\right),x_{m}\left(j\right)$) are considered similar or indistinguishable if the distance $\left(d_{ij}^{m}\right)$ between them is less than a tolerance parameter *r* in the multidimensional phase-space. In this sense, the pattern similarity is determined by the Heaviside function *Θ*($d_{ij}^{m}-r$) given in Equation (1), which acts like a two-state classifier that generates two possible categories: the patterns are similar or not.

(1) $$\Theta (d_{ij}^{m}-r)=\{\begin{array}{c} 1,\;if\;d_{ij}^{m}\;\leq \;r,\;\mathrm{patterns}\;\mathrm{are}\;\mathrm{similar}\\ 0,\;if\;d_{ij}^{m}\;>r,\;\mathrm{patterns}\;\mathrm{are}\;\mathrm{dissimilar} \end{array}.$$

The distance $d_{ij}^{m}$ is defined as:

(2) $$d_{ij}^{m}=max\left\{\left|(x\left(i\right)-x\left(j\right)\right|,\left|x\left(i-1\right)-x\left(j-1\right)\right|,\ldots ,\left|x\left(i-m+1\right)-x\left(j-m+1\right)\right|\right\},$$

namely the maximum absolute difference between the corresponding scalar components of the patterns $x_{m}\left(i\right)\;and\;x_{m}\left(j\right)$ (Chebyshev distance). According to [21], SampEn is defined as Equation (3):

(3) $$SampEn\left(m,r,N\right)=-ln\left(\frac{A_{r}^{m}}{B_{r}^{m}}\right),$$

where:

(4) $$B_{r}^{m}=\frac{1}{N-m}\overset{N-m}{\underset{i=1}{\sum }}\frac{1}{N-m-1}\overset{N-m}{\underset{j=1,j\neq i}{\sum }}\Theta \left(d_{ij}^{m}-r\right),$$

(5) $$A_{r}^{m}=\frac{1}{N-m}\overset{N-m}{\underset{i=1}{\sum }}\frac{1}{N-m-1}\overset{N-m}{\underset{j=1,j\neq i}{\sum }}\Theta \left(d_{ij}^{m+1}-r\right).$$

$B_{r}^{m}$ represents the probability that two patterns will match for *m* samples, and $A_{r}^{m}$ the probability that two patterns will match for *m* + 1 samples. The parameter *r* is usually set as a percentage of the standard deviation (SD) of the time series [22], which allows series with different amplitudes to be compared.

#### 2.2.2. FuzEn

Considering that in the real world the limits between categories may be ambiguous, thus making the decision on whether a pattern completely belongs to a specific category difficult, FuzEn employs a fuzzy membership function to obtain the degree of similarity between two patterns of length *m* [1,2]. The family of fuzzy functions should include the following characteristics: (i) continuous functions in order to avoid that the similarity change abruptly; (ii) convex functions to guarantee that self-similarity is the maximum. In FuzEn, the degree of similarity $D_{ij}^{m}=\lambda \left(d_{ij}^{m},n,r\right)$ between two patterns $x_{m}\left(i\right)$ and $x_{m}\left(j\right)$ is determined by the following fuzzy membership function [1,4]:

(6) $$\lambda \left(d_{ij}^{m},n,r\right)=\exp\left(\frac{-{\left(d_{ij}^{m}\right)}^{n}}{r}\right),$$

where $d_{ij}^{m}$ is the Chebyshev distance between patterns given in Equation (2), *r* is the tolerance parameter, and *n* defines the membership function shape. The membership function corresponds to a Gaussian function for *n* = 2, and to rectangular function for *n* = ∞. Similar to the definition of SampEn, the probability that two patterns $x_{m}\left(i\right)$ and $x_{m}\left(j\right)$ or $x_{m+1}\left(i\right)$ and $x_{m+1}\left(j\right)$ will match is given, respectively, as

(7) $$\varphi_{r}^{m}=\frac{1}{N-m}\overset{N-m}{\underset{i=1}{\sum }}\left(\frac{1}{N-m-1}\overset{N-m}{\underset{j=1,j\neq i}{\sum }}D_{ij}^{m}\right),$$

(8) $$\varphi_{r}^{m+1}=\frac{1}{N-m}\overset{N-m}{\underset{i=1}{\sum }}\left(\frac{1}{N-m-1}\overset{N-m}{\underset{j=1,j\neq i}{\sum }}D_{ij}^{m+1}\right).$$

Finally, FuzEn can be estimated by:

(9) $$FuzEn\left(m,r,N\right)=-\ln\left(\frac{\varphi_{r}^{m+1}}{\varphi_{r}^{m}}\right).$$

In this work, *n* = 2 was taken as a fixed parameter. It is important to mention that, although in previous works [1,2,4] $x_{m}\left(i\right)$ is generalized by removing a baseline, in the present study FuzEn was computed without removing a baseline of $x_{m}\left(i\right)$.

#### 2.2.3. Increasing Consistency of FuzEn Estimate

According to [9], although FuzEn offers a more accurate, more consistent, and less dependent on the data length entropy rate, the length of the series is still an important factor in the precision of FuzEn. In order to address this issue, authors in [9] presented new approaches to calculate FuzEn. These tried to improve FuzEn precision by increasing the number of patterns that are used in the computation, without changing the original length of the series. In this work, two of those approaches were taken into account: translated FuzEn (TFuzEn) and translated-reflected FuzEn (TRFuzEn).

TFuzEn calculates entropy rate in the same way as FuzEn but defines $x_{m}\left(i\right)$ by eliminating the mean value of the *m*-patterns, which can increase the number of similar patterns. The procedure to eliminate the baseline takes the vector *x_m_*(*i*) and subtracts from each component the temporal mean computed over the entire pattern as follows:

(10) $$x_{m}\left(i\right)=\left(x\left(i\right),x\left(i+1\right),\ldots ,x\left(i+m-1\right)\right)-\left(\mu \left(i\right),\mu \left(i\right),\ldots ,\mu \left(i\right)\right),$$

where μ(i) is defined as

(11) $$\mu \left(i\right)=\frac{1}{m}\overset{m-1}{\underset{j=0}{\sum }}x\left(i+j\right).$$

The second approach given in [9] involves one additional transformation over the *m*-dimensional patterns, in order to increase the matches of similar patterns. The additional transformation is “reflection” that implies to perform a reflection operation on $x_{m}\left(i\right)$, resulting in the reflected subsequence $x_{m}{}^{R}\left(i\right)$ over the translated pattern as follows:

(12) $$x_{m}{}^{R}\left(i\right)=\left(x\left(i+m-1\right),x\left(i+m-2\right),\ldots ,x\left(i+1\right),x\left(i\right)\right)-\left(\mu \left(i\right),\mu \left(i\right),\ldots ,\mu \left(i\right)\right),$$

leading to the elimination of the mean value of the reflected *m*-dimensional pattern and to the computation of TRFuzEn. 

#### 2.2.4. Eliminating Trends before FuzEn Computation

Inherent FuzEn (IFuzEn) [10] computes inherent functions, namely intrinsic mode functions (IMFs), obtained from the empirical mode decomposition (EMD), for eliminating superimposed trends in time series [23,24,25,26,27,28]. As superimposed trends in physiological signals is very common, and these trends could affect the estimation of entropy-based analysis by increasing the standard deviation of the signal, IFuzEn was proposed to increase the reliability of complexity evaluation in realistic EEG applications [10]. Indeed, before applying fuzzy entropy-based methods, IFuzEn implements a preprocessing stage to eliminate superimposed trends in the time series. In this work, FuzEn and TFuzEn were applied on time series after trend filtering, and they were represented as IFuzEn and ITFuzEn, respectively. The concepts about EMD and IMFs are described in Appendix A.

### 2.3. MSE and RMSE

RMSE is a technique based on the MSE approach [16], which applies SampEn as a function of time scale (TS) in order to perform a multiscale irregularity assessment. In order to do that, MSE follows the next three steps:

(a) Elimination of the fast temporal scales to focus on gradually slower time scales, applying a low-pass finite impulse response (FIR) filter. This FIR filter is based on the average of *TS* samples, as it is indicated in Equation (13):
  (13) $$x^{TS}\left(j\right)=\frac{1}{TS}\overset{TS-1}{\underset{k=0}{\sum }}x\left(j-k\right),\;1\leq j\leq N,$$
  where $x^{TS}\left(j\right)$ represents the original series x(i) filtered at the time scale *TS*. This filter has (i) slow roll-off of the main lobe; (ii) large transition band; (iii) important side lobes, and; (iv) cutoff frequency $f_{c}=0.5/TS$ cycles per sample.
(b) Downsampling the filtered series $x^{TS}\left(j\right)$ by the scale factor *TS*, so that $x^{TS}\left(j\right)$ has the same time duration of x(i) but with a smaller number of samples as a function of the factor *TS*. Due to the large transition band and the important side lobes, the FIR filter given in Equation (13) is inefficient to prevent aliasing when the filtered series are downsampled [11], and therefore, signals with high-power frequency components, near the center frequency of 0.25 cycles per sample, could generate artifactual components in the downsampled signals.
(c) Calculation of the SampEn in each filtered series $x^{TS}\left(j\right),$ according to the Section 2.2.1. In MSE, the tolerance parameter *r* is fixed as a percentage of the SD of the original series x(i) (usually 15%) and it is kept constant for all the series $x^{TS}\left(j\right)$. Due to that, and considering that the SD of filtered series is reduced by the low-pass filtering procedure, MSE measures not only the variations of signals regularity with *TS* but also the variations in the SD of the series $x^{TS}\left(j\right)$.

RMSE introduces two substantial variations in relation with MSE:

(a) The suboptimal FIR filter of MSE is substituted with a sixth order low-pass Butterworth filter to obtain the filtered series $x^{TS}\left(j\right)$. This Butterworth filter has the following characteristics: (i) flat response in the pass band; (ii) faster roll-off; (iii) no side lobes in the stop band, and; (iv) cutoff frequency $f_{c}=0.5/TS$ cycles per sample. This filter, in comparison with the FIR filter given in Equation (13), limits as much as possible the aliasing for any TS during downsampling.
(b) The tolerance parameter *r*, used for comparing patterns, is updated for each time scale according to an assigned fraction of the SD of each filtered series $x^{TS}\left(j\right)$. Therefore, RMSE does not depend on the reduction, generated by the low-pass filtering procedure, of the SD of the $x^{TS}\left(j\right)$. We make reference to [11] for specific details on the method.

In the present study, RMSE was applied as a technique of multiscale analysis according to (i) the original definition of RMSE (i.e., using a low-pass Butterworth filter, downsampling, and applying SampEn with tolerance parameter *r* updated for each time scale over the filtered series), and; (ii) replacing SampEn with FuzEn approaches as FuzEn, TFuzEn, TRFuzEn, IFuzEn, and ITFuzEn. RMSE in its several variants was computed at different *TS* of the proposed synthetic and experimental time series. According to previous works [11,12,13,14,15,16], in this study *r* = 0.15 × SD and *m* = 2 were taken as fixed parameters (in SampEn and FuzEn approaches) to compare RMSE values of the synthetic series for different lengths *N*. In order to evaluate how the tolerance parameter *r* affects the performance of RMSE values of the EEG signals, this parameter was also varied between 0.10 and 0.30 in steps of 0.05.

The different time scales in RMSE can be associated with the traditional EEG-frequency bands δ, θ, α, and β. Indeed, since EEG signals were resampled to 128 Hz, the time scale *TS* = 1 corresponds to the original EEG signal (with theoretical frequencies from 0 to Nyquist frequency ($f_{N})$, i.e., 128/2 = 64 Hz), *TS* = 2 contains frequencies from 0 to $f_{N}$/*TS* = 64/2 = 32 Hz, *TS* = 3 contains frequencies from 0 to $f_{N}$/3 = 21.3 Hz, and so on. Therefore, the following approximate association between EEG-frequency bands and *TS* was done: (i) β band corresponds to 2 ≤ *TS* ≤ 5; (ii) α band corresponds to 5 ≤ *TS* ≤ 8; (iii) θ band corresponds to 8 ≤ *TS* ≤ 16, and; (iv) δ band corresponds to *TS ≥ 16*.

#### Statistical Analysis

In this work, synthetic time series x(i) (see Section 2.1.1) of *N* = 10,000, *N* = 1000, and *N* = 100 were analyzed with RMSE. Time scales varied from *TS* = 1 to *TS* = 20. Bearing in mind that the length of the filtered series $x^{TS}\left(j\right)$ is reduced by the scale factor *TS*, the shorter series (with *TS* = 20) for *N* = 10,000 was of 500 samples, for *N* = 1000 was of 50 samples, and for *N* = 100 was of five samples. Consistency of RMSE values, in relation to the length *N* of the series, was evaluated graphically. In this sense, the RMSE metric was consistent whether the plots of RMSE values in different synthetic time series held the same relative behavior for different values of *N*. In other words, if the RMSE values of a time series $x_{1}\left(i\right)$ were higher than the RMSE values of a time series $x_{2}\left(i\right)$, for a specific *TS* and length *N*, that situation had to be maintained for the same *TS* at different values of *N*.

The prediction probability score (Pk) was applied to measure how well RMSE of EEG signal predicted the pain response of the patients. The Pk was proposed in [29] as statistical measurement to assess the performance of anesthetic depth indicators. Indeed, given two random data points with different observed anesthetic states, Pk is the probability that the values of a monitor or indicator in those data points predict correctly the observed anesthetic states, namely.

(14) $$Pk=\frac{P_{c}+P_{tx}/2}{P_{c}+P_{d}+P_{tx}},$$

where $P_{c}$, $P_{d}$, and $P_{tx}$ are the respective probabilities of concordance, discordance or x-only tie, between the values of an indicator and the observed anesthetic states. Pk values ranges from 0 to 1, where (i) Pk = 0.5 represents a complete randomness (concordance equal to discordance); (ii) 0.5 < Pk < 1, concordance is more likely than discordance; (iii) Pk = 1 corresponds to perfect concordance ($P_{d}$ and $P_{tx}$ are both equal to zero); (iv) 0 < Pk < 0.5, concordance is less likely than discordance; (v) Pk = 0 means perfect discordance ($P_{c}$ and $P_{tx}$ are both equal to zero). 

In this work, EEG segments were classified according to the noxious stimuli applied to the patient during the USE procedure, as follows (see Table 1):

(a) Response after a firm nail-bed pressure: (i) group 2 ≤ RSS ≤ 5, which included patients that moved (feel pain) in response to the noxious stimuli; (ii) group with RSS = 6, which did not move in response to the noxious stimuli.
(b) Response after endoscopy tube insertion: (i) group with GAG = 1, which felt pain; (ii) group with GAG = 0, which did not feel pain.

## 3. Results

### 3.1. RMSE of Synthetic Time Series

RMSE, using SampEn, FuzEn, TFuzEn, TRFuzEn, IFuzEn, and ITFuzEn, was calculated on all the realizations of the synthetic time series that were defined in Section 2.1.1. For this analysis, *r* = 0.15 × SD and *m* = 2 were taken as fixed parameters in SampEn and FuzEn approaches in order to compare RMSE values of the synthetic series for different lengths *N*.

**Figure 1:**
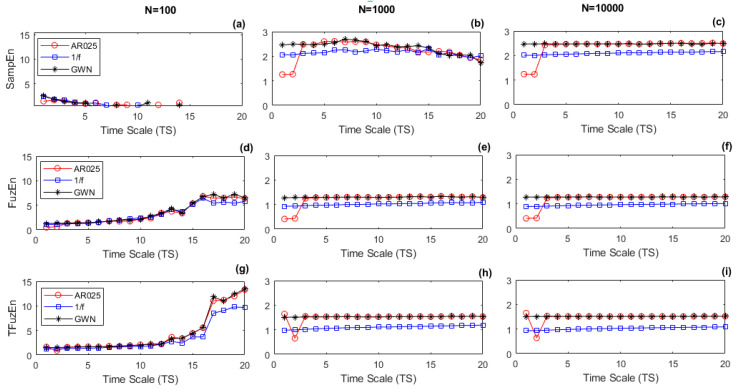

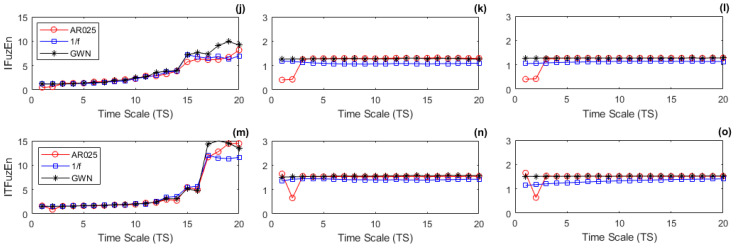
Multiscale analysis with refined multiscale entropy (RMSE), using sample entropy (SampEn) (**a**–**c**), fuzzy entropy (FuzEn) (**d**–**f**), translated FuzEn (TFuzEn) (**g**–**i**), inherent FuzEn (IFuzEn) (**j**–**l**), inherent translated FuzEn (ITFuzEn) (**m**–**o**), from 60 realizations of the type-1 simulated series (Gaussian white noise (GWN), 1/f, and AR025) for length *N* = 100 (left column), 1000 (middle column), and 10,000 (right column). In each case, the length of the simulated series was cropped up to the *N*th sample.

Figure 1 shows, for *N* = 100, *N* = 1000, and *N* = 10,000, the course of RMSE metrics as a function of TS. derived from the 60 realizations of the type-1 simulated series: GWN (black line with asterisk marker), 1/f (blue line with squared marker), and AR025 (red line with circle marker). In this analysis, the performance of RMSE for each simulated series was tested, including changes in the time series length *N*. As the courses of RMSE for TFuzEn and TRFuzEn were very similar, the last one was not included in Figure 1.

We considered the course of RMSE with SampEn and *N* = 10,000 as a reference (Figure 1c). The panel of Figure 1c shows that (i) the course was flat in the case of GWN; (ii) it had a slow but progressive increase in the case of 1/f noise, and; (iii) it was low and almost constant in short time scales (*TS* = 1–2) and rapidly increased in *TS* = 3, reaching a plateau in the case of AR025 process. From the comparison of Figure 1c with the other cases that are plotted in Figure 1, it was observed that:

(a) at *N* = 10,000, FuzEn (Figure 1f), and IFuzEn (Figure 1l) had a similar behavior of SampEn (Figure 1c) for all the synthetic time series, but the courses of TFuzEn (Figure 1i) and ITFuzEn (Figure 1o) showed a different behavior for the AR025 series, particularly in *TS* = 1 where the entropy value was higher than *TS* = 2, presenting a kind of ripple.
(b) at *N* = 1000, SampEn (Figure 1b) lost consistency in long scales (*TS* ≥ 10), which was evidenced specially in GWN and AR025 signals where the entropy value, that was higher than 1/f signal for *N =* 10,000, now was equal or lower that 1/f signal for *N* = 1000. On the contrary, all the fuzzy approaches (Figure 1e,h,k,n) showed a relative consistency for all the synthetic series at any time scale *TS*. TFuzEn (Figure 1h) and ITFuzEn (Figure 1n) continued showing a kind of ripple for the AR025 series between *TS* = 1 and *TS* = 2;
(c) at *N* = 100, all the RMSE metrics lost consistency. Although SampEn values (Figure 1a) could not be obtained for time scales *TS* ≥ 7 (short series with less than 14 samples), all the fuzzy approaches could be computed for *TS* values between 1 and 20.

**Figure 2:**
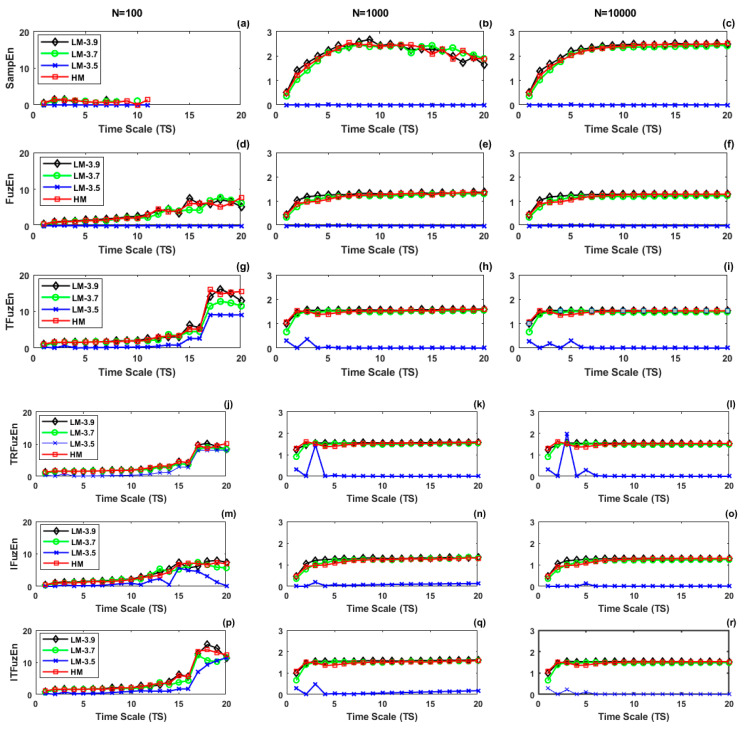
Multiscale analysis with RMSE, using SampEn (**a**–**c**), FuzEn (**d**–**e**), TFuzEn (**g**–**i**), anslated-reflected FuzEn (TRFuzEn) (**j**–**l**), IFuzEn (**m**–**o**), and ITFuzEn (**p**–**r**), from 30 realizations of the type-2 simulated series (LM-3.5, LM-3.7, LM-3.9, and Henon map (HM)) for length *N* = 100 (left column), 1000 (middle column), and 10,000 (right column).

Figure 2 shows, for *N* = 100, 1000, and 10,000, the course of RMSE approaches as a function of TS derived from the 30 realizations of the type-2 synthetic series: Logistic Map with a=3.5 (LM-3.5, blue line with asterisk marker), a=3.7 (LM-3.7, pink line with circle marker), a=3.9 (LM-3.9, black line with diamond marker), and Henon Map with α=1.4 and β=0.3 (HM, red line with square marker).

We considered the course of RMSE with SampEn and *N* = 10,000 as a reference (Figure 2c). The panel of Figure 2c shows that (i) the course was flat at zero value for the LM-3.5 signal, which corresponds to the Logistic map in oscillation condition (totally predictable signal) and, therefore, it was expected to have a low value of entropy; (ii) the course exhibited an initial increase at short time scales (1 ≤ *TS* ≤ 7) and reached a plateau at long time scales for LM-3.7, LM-3.9, and HM signal, which are signals belonging to Logistic and Henon maps in chaotic conditions. From the comparison of Figure 2c with the other cases that are plotted in Figure 2, it was observed that:

(a) at *N* = 10,000: (i) although all the fuzzy approaches (Figure 2f,i,l,o,r) had a similar behavior of SampEn (Figure 2c) for the chaotic series (LM-3.7, LM-3.9, and HM signals), i.e., the courses exhibited an initial increase and then reached a plateau, the entropy values were lower and with smaller span than SampEn; (ii) only FuzEn (Figure 2f) had a similar behavior than SampEn in the totally predictable signal (LM-3.5); (iii) the course for LM-3.5 showed ripples in TFuzEn (Figure 2i), TRFuzEn (Figure 2l), IFuzEn (Figure 2o) and ITFuzEn (Figure 2r), being TRFuzEn the one with the most prominent ripple;
(b) at *N* = 1000: (i) SampEn (Figure 2b) lost consistency in long scales (*TS* ≥ 10), which was evidenced specially in the LM-3.9 signal where the entropy value, that was higher or equal than the other signals for *N =* 10,000, now was lower than LM-3.7 and HM signals at long scales for *N* = 1000; (ii) all the fuzzy approaches (Figure 2e,h,k,n,q) showed a relative consistency for all the synthetic series at any time scale *TS*; (iii) only SampEn and FuzEn (Figure 2e) showed zero value for LM-3.5 in all the time scales; (iv) TFuzEn (Figure 2h), TRFuzEn (Figure 2k), IFuzEn (Figure 2n), and ITFuzEn (Figure 2q) continued showing a ripple in the course of LM-3.5;
(c) at *N* = 100: (i) entropy values decreased in long time scales for SampEn (Figure 2a), while entropy values in fuzzy approaches tended to increase in long time scales; (ii) only SampEn and FuzEn (Figure 2d) showed a zero value for LM-3.5 in all the time scales; (iii) SampEn values could not be obtained for time scales *TS* > 4 (short series with less than 25 samples) in LM-3.9 and for *TS* > 10 for the other series, but all the fuzzy approaches could be computed for all the *TS* values.

### 3.2. RMSE of EEG Signals

**Figure 3:**
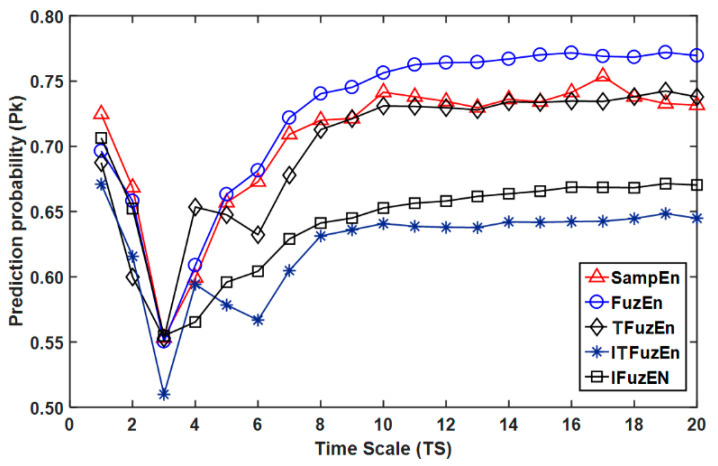
Prediction probability (Pk values) of the RMSE metrics, using SampEn, FuzEn, TFuzEn, IFuzEn, and ITFuzEn, for nociception assessment (2 ≤ Ramsay sedation scale (RSS) ≤ 5 vs. RSS = 6) after a firm nail-bed pressure. Pk = 0.5 represents a complete randomness and Pk = 1 a perfect prediction.

**Figure 4:**
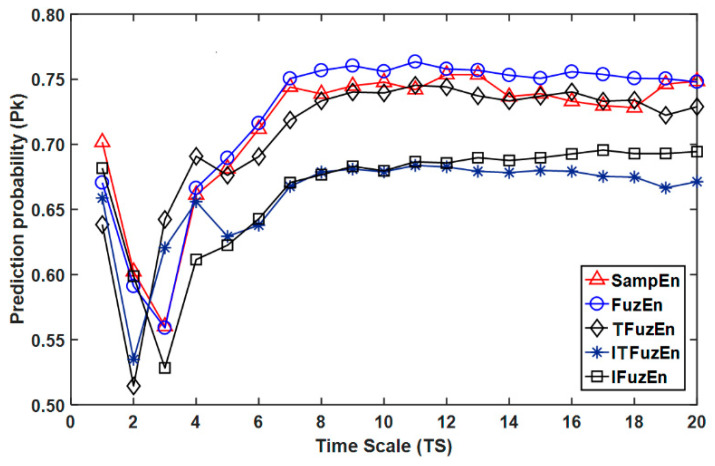
Prediction probability (Pk values) of the RMSE metrics, using SampEn, FuzEn, TFuzEn, IFuzEn, and ITFuzEn, for nociception assessment (gag reflex (GAG) = 1 vs. GAG = 0) after endoscopy tube insertion. Pk = 0.5 means a complete randomness and Pk = 1 a perfect prediction.

**Figure 5:**
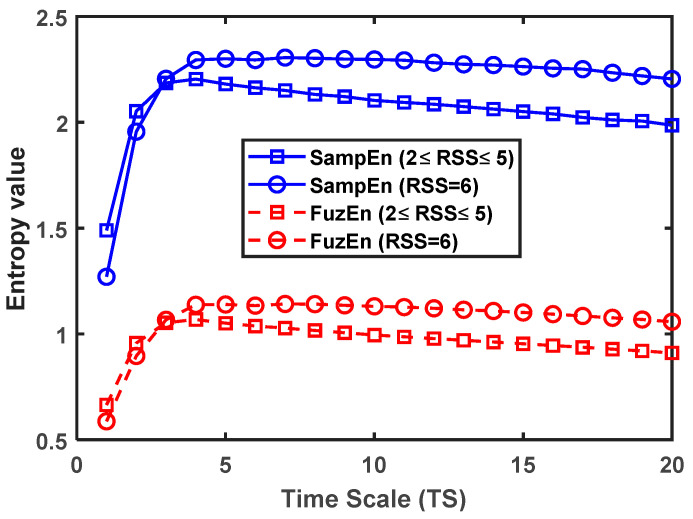
Mean values of the SampEn (blue-continuous lines) and FuzEn (red-dotted lines) as a function of the time scales *TS*, obtained from the electroencephalogram (EEG) segments in responsive states 2 ≤ RSS ≤ 5 (square marker), and; unresponsive state RSS = 6 (circle marker).

**Figure 6:**
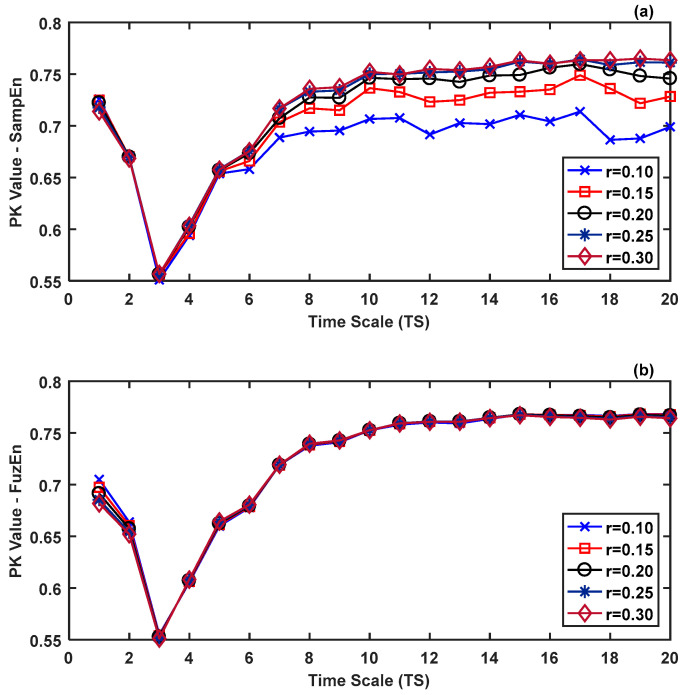
Pk values as a function of the time scales *TS*, for different values of the tolerance parameter *r* (0.10, 0.15, 0.20, 0.25, and 0.30). Results were obtained comparing responsive states (2 ≤ RSS ≤ 5) vs. unresponsive state (RSS = 6), using RMSE with (**a**) SampEn; (**b**) FuzEn.

Firstly, *N* = 6400, *r* = 0.15 × SD and *m* = 2 were taken as fixed parameters in SampEn and FuzEn approaches, in order to compare the prediction probability (Pk values) of the RMSE metrics for nociception assessment, using EEG signals (Figure 3, Figure 4 and Figure 5). Secondly, the tolerance parameter *r* was varied between 0.10 and 0.30, in steps of 0.05, to evaluate how this parameter affects the performance of RMSE metrics in the nociception assessment (Figure 6).

Figure 3 and Figure 4 show, as a function of time scales *TS*, the Pk values that were obtained to predict groups 2 ≤ RSS ≤ 5 vs. RSS = 6 and GAG = 1 vs. GAG = 0, respectively, using RMSE with SampEn and FuzEn approaches. As the courses of Pk values for TFuzEn and TRFuzEn were very similar, the last one was not included in Figure 3 and Figure 4. In both figures it is shown that:

(i) For long scales (6 ≤ *TS* ≤ 20), FuzEn, followed by SampEn, had the best Pk values, while ITFuzEn and IFuzEn had the worst performance.
(ii) For short scale (*TS* = 1), the best Pk value was for SampEn, followed by IFuzEn and FuzEn. The lowest Pk values were obtained at *TS* = 3 for all the approaches in Figure 3 (2 ≤ RSS ≤ 5 vs. RSS = 6), while, for Figure 4 the lowest Pk were at *TS* = 2 for ITFuzEn, TFuzEn and TRFuzEn, and at *TS* = 3 for SampEn, FuzEn, and IFuzEn.
(iii) When the Pk values were computed using FuzEn, the highest Pk values were obtained in time-scales larger than *TS* = 10. Since EEG signals were resampled to 128 Hz, time scales between *TS* = 10 and *TS* = 20 represent a EEG signal with frequency components that reduces the superior limit of the pass-band from 6.4 (*TS* = 10) to 3.2 Hz (*TS* = 20), gradually removing contributions in the β (14–30 Hz), α (8–14 Hz), and θ (4–8 Hz) bands of the EEG, and leaving the fluctuations in the δ (0.1–4 Hz) band of the EEG.

Since FuzEn and SampEn had the best Pk values at long time scales, Figure 5 was designed to show the mean values of the SampEn and FuzEn as a function of the time scales *TS*, obtained from the EEG segments divided into responsive (2 ≤ RSS ≤ 5) vs. unresponsive (RSS = 6) classes. It was observed that:

(i) SampEn showed higher entropy values than FuzEn in all the scales for both groups (2 ≤ RSS ≤ 5 and RSS = 6).
(ii) The courses of RMSE had a similar behavior in both metrics (SampEn and FuzEn) with an initial increase at short time scales (1 ≤ *TS* ≤ 3), a maximum near to *TS* = 4, and, then, a slow decrease at long time scales.
(iii) In both metrics (SampEn and FuzEn), the entropy value was higher in responsive than in unresponsive state at short scales (1 ≤ *TS* ≤ 2), but this situation changed at longer scales (4 ≤ *TS* ≤ 20), i.e., the entropy value was lower in responsive than in unresponsive state.

Figure 6 shows the Pk values as a function of the time scales *TS*, that were obtained to predict groups 2 ≤ RSS ≤ 5 vs. RSS = 6. Different values of the tolerance parameter *r* (0.10, 0.15, 0.20, 0.25, and 0.30) were considered, using RMSE with (a) SampEn; (b) FuzEn. It was observed that:

(i) When SampEn was computed (Figure 6a), the Pk values were almost equal at short scales (1 ≤ *TS* ≤ 5) for all the values of the parameter *r*, but not for long scales.
(ii) When FuzEn was computed (Figure 6b), the Pk values were practically equal for all the time scales *TS* and for all the values of the parameter *r*.
(iii) The best Pk values were obtained for long scales in both RMSE using SampEn and using FuzEn.

## 4. Discussion

In previous works [1,2], FuzEn has been proposed as an entropy measure that is more consistent and less dependent on the data length than SampEn, and several variants have been designed to further improve FuzEn performance over short time series. Indeed, approaches as TFuzEn, TRFuzEn, IFuzEn, and ITFuzEn have been introduced [9,10] to increase the number of patterns that are compared without changing the length of the time series and limiting the effect of local variation of the mean. In the present work, these metrics were included in a multiscale analysis, using RMSE applied to signals with different characteristics (fully stochastic, stochastic with long-range correlation, stochastic with some deterministic parts and thus partially predictable, totally predictable, chaotic and real EEG signals). The study was mainly focused to compare the performance of RMSE, with SampEn and FuzEn approaches, as a function of the length of the data and the type of the series. Additionally, metrics were compared according to the prediction probability value (Pk) of pain response in patients under sedation-analgesia, while varying the tolerance parameter *r*.

In relation with long data series (*N* = 10,000), the results showed that the course of RMSE had the same tendency with FuzEn approaches as with SampEn, when they were applied to signals with the following behavior (Figure 1 and Figure 2): fully stochastic (GWN), stochastic with long-range correlation (1/f), and chaotic (LM-3.7, LM-3.9, and HM). However, there were important differences between some of the RMSE courses obtained from partially predictable (AR025) and totally predictable (LM-3.5) signals. The more relevant case was the one related to the totally predictable (LM-3.5) signal. LM-3.5 is a periodic signal with a deterministic behavior, which should have a very low entropy rate value resulting from its periodic nature that does not generate new information. However, all the FuzEn variants, with the exception of FuzEn, showed ripples with values different from zero in this type of signal. This suggests that metrics such as TFuzEn, TRFuzEn, IFuzEn, and ITFuzEn introduce some kind of irregularity with different levels of predictability, especially in the longer time scales. Approaches as TRFuzEn and ITFuzEn are based on TFuzEn [9], so that all of them remove the mean value of *m*-dimensional patterns before computing the probability of finding matched patterns, thus suggesting that removing the mean over patterns might be responsible for the introduction of some degree of randomness.

Comparing the performance of RMSE courses as a function of the series length *N*, SampEn metric lost consistency using series with length *N* = 1000 in comparison to series of length *N* = 10,000. On the contrary, all the fuzzy approaches showed a relative consistency for all the synthetic series at any time scale *TS*, demonstrating to be less dependent on the data length as it was indicated in [1,2]. The loss of consistency in SampEn was more evident for long scales (*TS* ≥ 10), where the series has reduced its length to *N*/*TS* = 1000/10 = 100 samples or less. This result is in agreement with [21], where it was reported the dependence of SampEn with the series length and how this metric loses consistency when data length is smaller than 300.

The analysis with series of length *N* = 100 allowed the performance of RMSE metrics in short time series to be evaluated. Although all the metrics lost consistency compared to series with length *N* = 10,000, the most relevant finding was that, while SampEn values could not be obtained for long time scales, fuzzy approaches could be computed for all the *TS* values (from 1 to 20). For example, in this study, SampEn could not be computed for short series with less than 25 samples in LM-3.9 signals. By its definition, SampEn depends of the logarithm of the ratio ($A_{r}^{m}/B_{r}^{m}$), and this logarithm is indeterminate if there are not patterns that match for *m* and *m* + 1 samples at the same time. This condition is minimized in Fuzzy entropy definition and in its variants that increment the number of patterns that are compared without changing the length of the time series, allowing the computation of the metrics even in very short series.

About the nociception assessment using EEG signals for classifying RSS values and GAG responses (2 ≤ RSS ≤ 5 vs. RSS = 6 and GAG = 1 vs. GAG = 0 in Figure 3 and Figure 4, respectively), the best performance was obtained with FuzEn, followed by SampEn, in middle and long scales. These results suggest that although the variants of FuzEn are metrics less dependent on the data length, this does not mean that they provide an estimate of conditional probability better than SampEn or FuzEn. The methods used for these metrics to create new patterns increase the probability of finding similar patterns, thus increasing the regularity of the series and reducing conditional entropy, and this attitude reduces the possibility to differentiate nociceptive states. Considering that the procedure to compute RMSE reduces the time series length as a function of *TS*, the best Pk values that were obtained with FuzEn can be related with the fact that FuzEn was more consistent and less dependent on the data length than SampEn. On the other hand, ITFuzEn and IFuzEn had the worst performance in middle and long scales. These two approaches are based on the EMD as a method to reduce superimposed trends in time series, in order to moderate the effect of trends in the increment of the standard deviation of the data. However, this procedure seems to worsen the performance of RMSE in middle and long scales in comparison with approaches that do not eliminate the trends as SampEn. This can be related with the fact that RMSE adjusts the tolerance for comparing patterns as function of the standard deviation of each the time series, thus limiting the dependence of RMSE on the reduction of variance due to the elimination of the fast temporal scales [11].

In relation to the results of RMSE obtained from the EEG segments when they were divided into responsive (2 ≤ RSS ≤ 5) and unresponsive (RSS = 6) classes (Figure 5), we conclude that (i) the lower values of FuzEn compared to SampEn are linked to the fact that the fuzzy membership functions increases the probability of finding similar patterns that match for *m* and *m* + 1 samples at the same time, thus increasing the regularity in the series; (ii) the regularity of the EEG signal was higher at short time scales (*TS* < 3) than in long time scales, which indicates that the lower frequency bands as α, θ and δ contain the more complex activity of the EEG in those patients; (iii) the complexity of the EEG segments relevant to the responsive class was higher at short time scales (*TS* = 1) and lower at long time scales than those relevant to the unresponsive class. As was discussed in [15], the scalp and facial muscle activity, in patients of the responsive group (patients with low sedation level), is associated to the higher complexity of the EEG in short time scales and to a greater probability of feel pain than the unresponsive group. At long time scales, the higher complexity in unresponsive group (patients with higher sedation level than the responsive group) is associated with the displacement of the EEG activity to low frequency bands as the level of sedation increases.

The impact of the tolerance parameter *r*, used to determine the similarity between patterns in SampEn and FuzEn, on the Pk statistic was evaluated. Indeed, the Pk was computed for different values of *r*, between *r* = 0.10 and 0.30, for each one of the RMSE metrics as a function of the *TS*, in relation to the ability of predicting responsive (2 ≤ RSS ≤ 5) and unresponsive (RSS = 6) subjects. At long time scales, RMSE with SampEn showed important variations in the Pk statistic for different *r* values at the same *TS*, while the Pk was practically equal in FuzEn for all the *r* values at the same *TS*. This multiscale analysis evidenced the low dependence of FuzEn with the value of the tolerance parameter *r*, in comparison with SampEn. As was pointed out in [1,2], the Heaviside function used by SampEn creates a rigid boundary that can lead to sudden variations of the entropy values when the tolerance parameter *r* varies. As a result, SampEn may rise or fall dramatically when the tolerance *r* is slightly changed. Conversely, FuzEn employs an exponential function with soft boundaries, in such a way that entropy values are more stables to changes in *r*.

## 5. Conclusions

This work validated the RMSE performance when FuzEn, with different refinements, was implemented instead of SampEn for computing the entropy-based measure. Indeed, SampEn, FuzEn, TFuzEn, TRFuzEn, IFuzEn, and ITFuzEn were applied at different time scales to synthetic and experimental time series. The results of the present study suggest that it is necessary to be cautious with the application of some FuzEn variants, and with the interpretation of their findings. Indeed, approaches based on the elimination of the mean value of the patterns before computing the probability of finding matches (TFuzEn, TRFuzEn, and ITFuzEn) showed high entropy values over predictable process that should have low entropy values. Additionally, FuzEn methods using the EMD approach to reduce the effect of superimposed trends in time series (ITFuzEn and IFuzEn) seem to worsen the performance of RMSE at middle and long scales. In general, FuzEn showed a similar behavior to SampEn in series with long lengths, with the advantage of being more consistent than SampEn over short-length time series, less dependent on the tolerance parameter *r*, and stronger in the nociception prediction especially at long time scales (6 ≤ *TS* ≤ 20). Therefore, because of that, FuzEn can be more suitable in real-time and real-world applications.

## Figures and Tables

**Table 1:** Observed categorical responses in the database.

Groups	Score	Description	No. EEG Windows
2 ≤ RSS ≤ 5	RSS = 2	The patient is awake, quiet and cooperative	422
	RSS = 3	The patient is drowsy but responds to commands	641
	RSS = 4	The patient is asleep with brisk response to stimulus	428
	RSS = 5	The patient is asleep with sluggish response to stimulus	360
RSS = 6		No response (absence of movement) to firm nail-bed pressure.	782
GAG = 0		Absence of nausea reflex after endoscopy tube insertion	411
GAG = 1		Presence of nausea reflex after endoscopy tube insertion	125

No. EEG windows: number of EEG segments with a duration between 50 and 60 s recorded just before the response annotation according to RSS or GAG classification.

**Figure A1:**
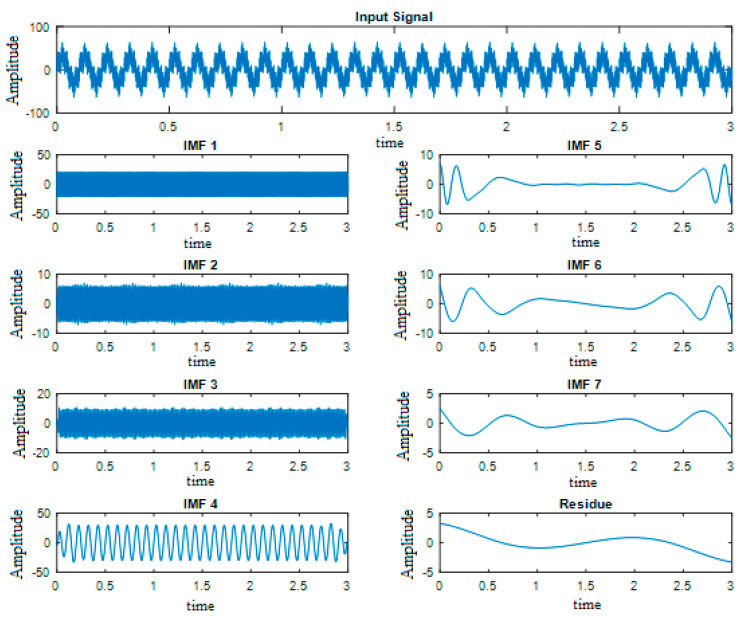
Empirical mode decomposition (EMD) results on a sinusoidal input signal with five frequency components: 10, 50, 100, 300, and 500 Hz, with an amplitude of 30, 10, 1, 20 and 5, respectively.

**Figure A2:**
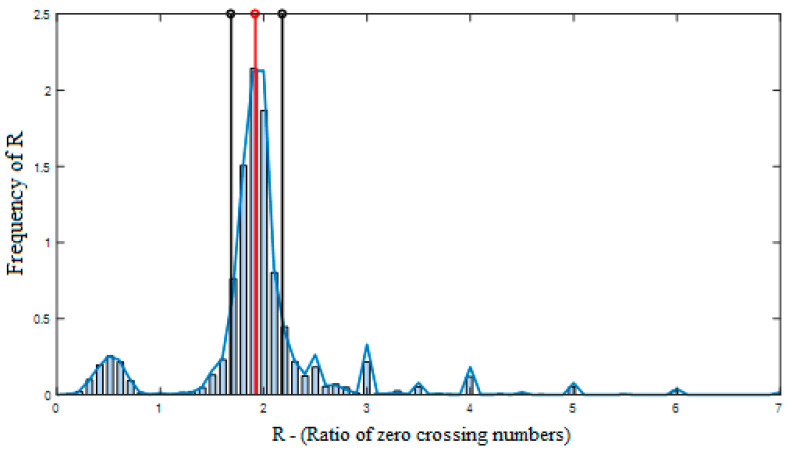
Ratio approach: empirical distribution of *R* vectors of EEG signals used in this work. The vertical red line represents the mean of the distribution of the *R* vectors ($R_{i}\approx 2$). Vertical black lines represent the left and right thresholds using a value of p=15.

**Figure A3:**
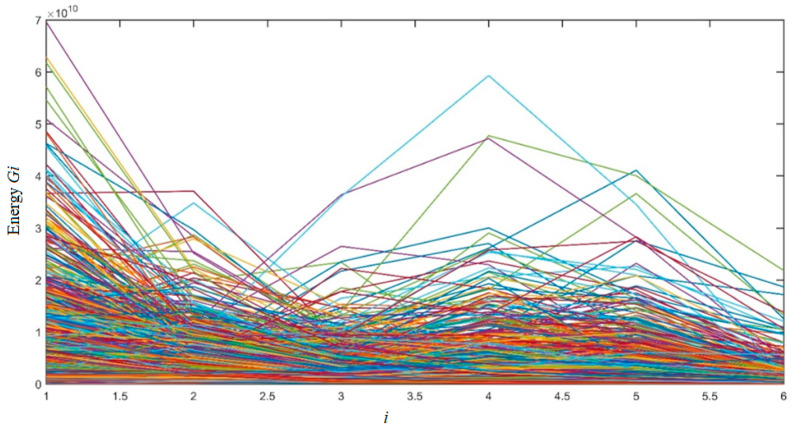
Energy approach: G vectors of the EEG signals used in this work.

**Figure A4:**
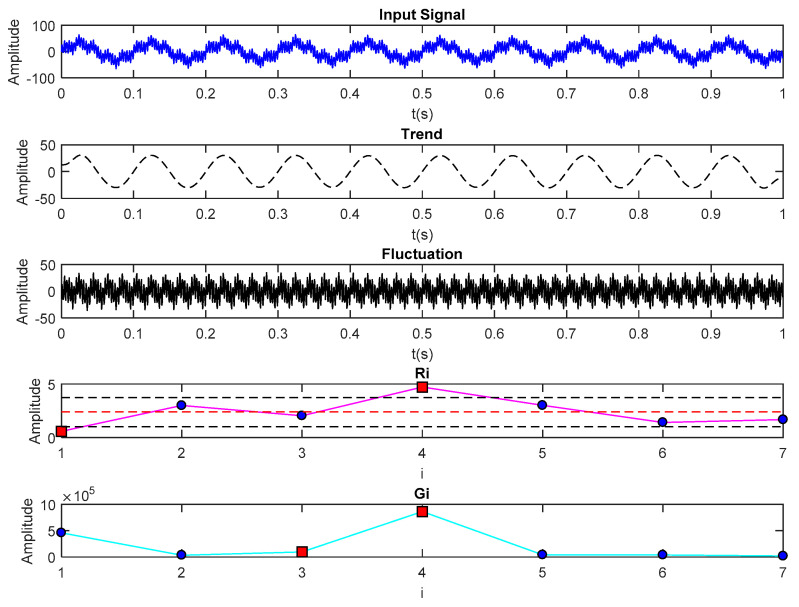
Trend filtering process applied on a sinusoidal input signal with five frequency components.

## Appendix A. Empirical Mode Decomposition (EMD)

The empirical mode decomposition (EMD) algorithm decomposes a time series into a series of intrinsic mode functions (IMFs), which are simple oscillatory functions with varying amplitude and frequency [23,24,25]. Given a time signal x(t), with an initialization step i=1 and $\rho^{0}=x\left(t\right)$, the EMD obtains the IMFs of x(t) according to the following algorithm:

(1) Identify all the local extrema of the signal (local minima and local maxima of $\rho^{i-1}$).
(2) Interpolate between these local extrema, ending up with the upper and lower envelopes of $\rho^{i-1}$ ($E_{up}\left(t\right)$ and $E_{low}\left(t\right)$). In this case a cubic spline interpolation was used according with work in [25].
(3) Find the local trend as the average of the upper and lower envelopes, $Q_{i}\left(t\right)=(E_{up}\left(t\right)+E_{low}\left(t\right))/2$.
(4) Determine the local fluctuation as, $h\left(t\right)=X\left(t\right)-Q_{i}\left(t\right)$.
(5) Evaluate h(t) as a candidate of inherent functions; h(t) will be an IMF if it satisfies two conditions: (i) the number of extrema and zero crossings must be either equal or differ at most by one; (ii) the average value between the envelope defined by the local maxima and the envelope defined by the local minima, must be zero at any point [23,25].
(6) If h(t) is not an IMF, then go to step (1) with $\rho^{i-1}=h\left(t\right)$.
(7) If h(t) is an IMF, save it as the $i^{th}$ intrinsic mode function $m^{i}=h\left(t\right)$, and calculate the residue r=x(t)−h(t). Then, take $\rho^{i}=r$, increment i, and return to step (1).

The above algorithm halts when the residue becomes a monotonic function, it means, when residue has no additional oscillations (there are not more local maxima or minima), and no more IMF can be extracted [23,24,25,26,27,28]. In this decomposition, $m^{i}$ contains a ‘‘spectrum’’ of local oscillations in x(n), where $m^{1}$ corresponds to the highest frequency (shortest-period) oscillations and $m^{l}$, where l represents the largest index for which $m^{i}$ is defined, corresponds to the lowest frequency (longest-period) oscillations.

Figure A1 shows the EMD results on a sinusoidal input signal with five frequency components: 10, 50, 100, 300, and 500 Hz, with an amplitude of 30, 10, 1, 20 and 5, respectively.

If we sum all IMFs and residue, we will obtain the original input signal. This is,

(A1) $$x\left(n\right)=\left(\overset{l}{\underset{i=1}{\sum }}m^{i}\right)+r.$$

However, the main purpose of using EMD on our input signal is performing a trend filtering. This can be done choosing the best IMF index $i^{*}$ which allows to separate the trend from the fluctuation [25,26,27]. In the presence of a trend, the IMF index i shows a rupture in two properties; (i) the mean frequency of the successive IMFs of broadband processes decrease [24,25,26]; (ii) the ‘‘energy’’ of the IMFs decreases as the index of the IMFs increases [25,27,28]. Taking into account the described IMFs properties, the authors in [25] carried on a study to determine the best approach to estimate $i^{*}$. Results in [25] showed that the best one is the energy-ratio approach. This approach combines the energy approach and ratio approach in order to reduce the false detections of $i^{*}$.

The ratio approach depends on the zero crossing numbers of IMFs. Given a time series, the *i*th ratio of the zero crossing numbers is defined as $R_{i}=Z_{i-1}/Z_{i}$, where $Z_{i}$ is the zero crossing number of its *i*th IMF. The approximation $R_{i}\approx 2$ holds if the time series under study is a realization of a generic broadband process, as was observed in [24,25,26]. The approximation $R_{i}\approx 2$ fails for i close the best IMF index. Then, the best IMF index $i^{*}$ using ratio approach will be the smallest index i for which $R_{i}$ is ‘‘significantly different from 2’’. In order to support the above, in this work, we took 2633 EEG signals, each one with a length N = 6400 samples. We then computed the IMFs of each signal and found a vector R which contained the $R_{i}$ values. After, we plotted the empirical distribution of *R* vectors which is shown in Figure A2. From this figure it is possible to conclude that, effectively, the mean of the distribution is approximately 2, then $R_{i}\approx 2$ holds.

The energy approach is based on an empirical property of the so-called ‘‘energy’’ of the IMFs, which can be defined as:

(A2) $$G_{i}=\sum {\left|m_{i}\right|}^{2},1\leq i\leq l.$$

$G_{i}$ is a decreasing sequence in i if the time series under study are realizations of a generic broadband process [27,28]. Thus, the best IMF index $i^{*}$, using energy approach, will be the smallest index i for which $G_{i}$ increases (the smallest index i≥2 such that $G_{i}>G_{i-1}$). This was also supported using the same EEG signals as in the ratio approach. In this case, $G_{i}$ was calculated for each IMF of each signal, creating a G vector. All G vectors were plotted, as is shown in Figure A3.

Finally, the energy-ratio approach combines the energy approach and ratio approach, thus, the best IMF index $i^{*}$ using energy-ratio approach will be the smallest index i where the ratio approach as well as the energy approach are fulfilled.

Therefore, trend T(t) and fluctuation F(t) are defined as:

(A3) $$T\left(t\right)=\overset{l}{\underset{i=i^{*}}{\sum }}m_{i},$$

(A4) $$F\left(t\right)=\overset{i^{*}-1}{\underset{i=1}{\sum }}m_{i}.$$

An example of the above is presented in Figure A4.

Figure A4 shows the trend filtering process on the same signal used in Figure A1. Additionally, the plot of values is shown. In the $R_{i}$. plot, the mean value and lateral thresholds are represented with a red-dashed line and black-dashed lines, respectively. In this example, the best IMF index is *I* = 4, because, in this value, the ratio approach as well as energy approach are fulfilled.
